# Reducing the Number of Intrusive Memories of Work-Related Traumatic Events in Frontline Health Care Staff During the COVID-19 Pandemic: Case Series

**DOI:** 10.2196/55562

**Published:** 2024-11-18

**Authors:** Veronika Kubickova, Craig Steel, Michelle L Moulds, Marie Kanstrup, Sally Beer, Melanie Darwent, Liza Keating, Emily A Holmes, Lalitha Iyadurai

**Affiliations:** 1 Oxford Institute of Clinical Psychology Training and Research Oxford Health NHS Foundation Trust Oxford United Kingdom; 2 Department of Experimental Psychology University of Oxford Oxford United Kingdom; 3 School of Psychology UNSW Sydney Sydney Australia; 4 Department of Psychology Uppsala University Uppsala Sweden; 5 Behavioral Medicine Theme Women’s Health and Allied Health Professionals Karolinska University Hospital Stockholm Sweden; 6 Emergency Medicine Research Oxford Oxford University Hospitals NHS Foundation Trust Oxford United Kingdom; 7 University Department of Emergency Medicine Royal Berkshire NHS Foundation Trust Reading United Kingdom; 8 Department of Women’s and Children’s Health Uppsala University Uppsala Sweden; 9 Department of Psychiatry University of Oxford Oxford United Kingdom

**Keywords:** intrusive memories, digital intervention, psychological trauma, remote delivery, health care staff, COVID-19, case series

## Abstract

**Background:**

Frontline health care staff are frequently exposed to traumatic events as part of their work. Although this study commenced before the emergence of COVID-19, levels of exposure were heightened by the pandemic. Many health care staff members report intrusive memories of such events, which can elicit distress, affect functioning, and be associated with posttraumatic stress disorder symptoms in the long term. We need evidence-based interventions that are brief, preventative, nonstigmatizing, suitable for the working lives of frontline health care staff, and effective for repeated trauma exposure. A brief, guided imagery-competing task intervention involving a trauma reminder cue and Tetris gameplay may hold promise in this regard, given evidence that it can prevent and reduce the number of intrusive memories following trauma across various settings.

**Objective:**

This case series aims to investigate the impact of a brief imagery-competing task intervention on the number of intrusive memories, general functioning, and symptoms of posttraumatic stress, anxiety, and depression, and examine the feasibility and acceptability of the intervention for UK National Health Service frontline health care staff. The intervention was delivered with guidance from a clinical psychologist.

**Methods:**

We recruited 12 clinical staff from the UK National Health Service, specifically from emergency departments, the intensive care unit, and the ambulance service. We evaluated the intervention using an AB single-case experimental design, where the baseline (A) was the monitoring-only phase and the postintervention (B) period was the time after the intervention was first administered. Methods were adapted once the COVID-19 pandemic began.

**Results:**

There was a decrease (59%) in the mean number of intrusive memories per day from baseline (mean 1.29, SD 0.94) to postintervention (mean 0.54, SD 0.51). There was a statistically significant reduction in the number of intrusive memories from baseline to postintervention, as shown by an aggregated omnibus analysis with a small effect size (τ-*U*=–0.38; *P<*.001). Depression, anxiety, and posttraumatic stress symptoms all significantly reduced from preintervention to postintervention. Participants also reported improvements in functioning based on both quantitative and qualitative measures. The intervention was feasible to deliver and rated as acceptable by participants.

**Conclusions:**

These preliminary findings suggest that this brief therapist-guided imagery-competing task intervention offers a potential approach to mitigating the impact of work-related traumatic events in frontline health care staff, both during a pandemic and beyond. Randomized controlled trials will be an important next step.

## Introduction

### Background

Frontline health care staff, including emergency department (ED) staff, intensive care unit (ICU) staff, and paramedics, frequently encounter highly stressful and traumatic clinical events, such as patient death, resuscitations, and treating patients with severe injuries [1]. These experiences often lead to intrusive memories, which are involuntary sensory recollections, primarily visual but sometimes involving other senses, such as smell and sound [2-4]. These memories can evoke strong negative emotions [5], disrupt concentration [6], and impair daily functioning [7], making them a critical treatment target.

Intrusive memories are a core symptom of posttraumatic stress disorder (PTSD) and acute stress disorder [8], and they may drive posttraumatic stress symptomatology [9]. Reducing intrusive memories could lead to a broader reduction in PTSD symptoms [10], suggesting a potential “therapeutic cascade” [11].

PTSD rates were already high among National Health Service (NHS) health care staff before the COVID-19 pandemic and increased substantially during it, with prevalence ranging from 21% among health care workers across 21 countries to 40% among ICU staff in the United Kingdom [12-17]. Unrelenting workloads, burnout, and stress are major drivers of nurses leaving the profession [18]. Furthermore, investigating new initiatives to improve ED staff engagement, resilience, and retention has been highlighted as a research priority for emergency medicine in the United Kingdom [19]. Therefore, developing effective treatments to protect the mental well-being of frontline health care staff is essential [20].

Frontline health care staff may experience intrusive memories without meeting the full diagnostic criteria for PTSD. Many may prefer shorter, more targeted interventions due to the stigma surrounding mental health issues among health care professionals and the substantial workload imposed by the pandemic [21,22]. It is crucial to develop novel interventions that are effective, brief, accessible, scalable, and nonstigmatizing, and that can be used repeatedly to manage ongoing trauma exposure.

A novel, brief, low-intensity, imagery-competing task intervention has been developed to target intrusive memories of trauma [23-25]. The intervention, which lasts approximately 25 minutes, can be administered in a single researcher-assisted session and self-administered thereafter. It aims to reduce the number of intrusive memories by using principles from cognitive science, specifically dual tasking, the properties of mental imagery [26], and the theories of memory consolidation and reconsolidation [27].

The intervention’s mechanism involves performing a visuospatial task while the trauma memory is still labile. This is theorized to interfere with the memory consolidation process and prevent the emergence of intrusive memories [28]. This approach for recent trauma has been supported by 3 randomized controlled trials involving women who experienced traumatic childbirth [29], motor vehicle accident survivors in the ED [30], and a mixed trauma sample of individuals presenting to the ED [25]. In these trials, participants who received the intervention reported significantly fewer intrusive memories in the first week after exposure to trauma compared with control groups, with effect sizes ranging from small to medium (Cohen *d*=0.43 to Cohen *d*=0.67) [25,29,30]. In addition, the intervention significantly reduced intrusion-related distress and vividness [25].

Evidence also supports that the intervention can reduce older, established trauma memories, with the effect informed by theories of memory reconsolidation interference. Promising results, including a decreased frequency of intrusive memories and improvements in functioning, have been shown in case series involving refugees [31], inpatients with complex PTSD [32], and women with childhood trauma [33,34].

A digital version of the intervention, delivered remotely with researcher support, was tested among health care staff members during the COVID-19 pandemic in a pilot study and was found to be feasible and acceptable [35]. This brief remotely delivered digital intervention was tested in a randomized controlled trial with the UK NHS ICU staff during the COVID-19 pandemic [36,37]. Participants who received immediate access to the intervention (with an initial guided session) reported significantly fewer intrusive memories at week 4 compared with those with delayed access. Intrusive memories also significantly decreased at week 8 (postintervention) compared with week 4 (preintervention) in the delayed access arm [37]. Furthermore, the immediate (vs delayed) intervention group reported reduced PTSD symptoms, insomnia, anxiety, posttrauma distress, and burnout, and improved work functioning and general well-being. Participants found the intervention to be acceptable and safe. The study provided strong evidence of the intervention’s efficacy for reducing intrusive memories among this group of health care staff [36]. While the intervention has been tested among hospital staff, its effectiveness among prehospital staff, such as paramedics, who often face unpredictable, high-intensity trauma in chaotic and risk-prone settings, remains unknown [38-40].

### Objectives

Given these promising findings and the need for novel approaches to support health care personnel beyond ICU staff, our study aimed to test the efficacy, feasibility, and acceptability of the brief guided imagery-competing task intervention in a sample of frontline prehospital and hospital NHS health care staff exposed to traumatic events in the course of their work. The primary aim was to determine whether the intervention reduced the number of intrusive memories of work-related traumatic events. We predicted a significant decrease in the number of intrusive memories from baseline to postintervention. Secondary aims included assessing changes in intrusive memory characteristics (eg, vividness and distress), their impact on daily functioning (eg, sleep and concentration), and other mental health symptoms (eg, posttraumatic stress, depression, and anxiety). In addition, we evaluated the intervention’s feasibility and acceptability among NHS health care staff.

## Methods

### Participants

**Figure 1:**
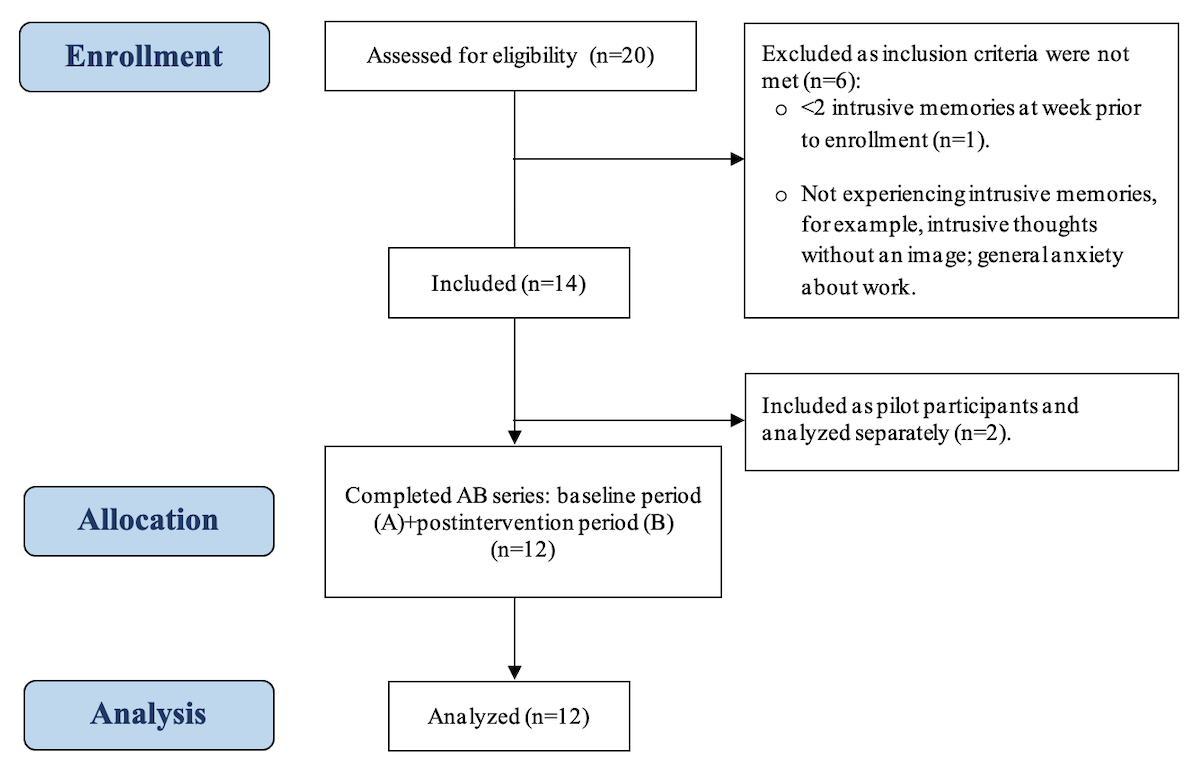
Adapted CONSORT (Consolidated Standards of Reporting Trials) flow diagram for the study.

In total, 12 frontline NHS health care staff members, including doctors, nurses, and paramedics, working in hospital (eg, ED and ICU) and prehospital departments (eg, ambulance services team) at Oxford University Hospitals, Royal Berkshire, and South Central Ambulance Service NHS Foundation Trusts participated in the study (recruited from February 2020 to September 2020). Inclusion criteria were aged ≥18 years; ability to read, write, and speak in English; being able and willing to provide informed consent and complete study procedures; employed as NHS hospital or prehospital clinical staff; having experienced at least 2 intrusive memories (self-rated as problematic) of a work-related traumatic incident in the previous 7 days; being able and willing to talk about the intrusive memories; ability to complete a web-based daily intrusive memory diary over a 2-to-3-week period; being capable of playing Tetris on a handheld device; and not currently undergoing treatment for PTSD. Participants were excluded if they had <2 intrusive memories per week during the baseline period or had started undergoing treatment for PTSD. The study was piloted on 2 participants (Multimedia Appendix 1). Figure 1 depicts an adapted CONSORT (Consolidated Standards of Reporting Trials) flow diagram for the study.

### Design

This case series used an AB single-case experimental design [41]. The primary outcome was the change in the number of intrusive memories of work-related traumatic events from baseline (A) to postintervention (B). The baseline was the period before the first administration of the intervention, that is, monitoring-only, and the postintervention period was anytime thereafter. The study consisted of a 1-week baseline period, a 2-week postintervention period following a single researcher-assisted intervention session, and a follow-up 4 weeks after the intervention session.

Following the single researcher-assisted intervention session, participants received instructions for using optional intervention boosters as needed in their daily lives. The intervention boosters could be researcher-assisted (ie, by scheduling a guided intervention session with the researcher) or self-administered (ie, playing Tetris for 25 minutes *immediately* after experiencing an intrusive memory, excluding a brief trauma reminder cue procedure required in the researcher-assisted intervention sessions). Participants were invited to use the intervention boosters for any new intrusive memories of work-related traumatic events they experienced after enrolling in the study; however, these were not included in the analysis. Participants could use the intervention boosters as many times as needed until week 4 (postintervention).

Due to COVID-19-related national restrictions, all participant interactions and intervention sessions were conducted remotely (ie, via videocall).

### Procedure

#### Training to Deliver the Intervention

To ensure proper intervention delivery and protocol adherence, the primary researcher, VK (a trainee clinical psychologist in the final year of clinical training), received comprehensive training, feedback, and monitoring from clinical psychologists experienced in delivering the intervention. This training included a 1-day web-based workshop covering the theoretical foundations, key components, and wider protocol aspects of the intervention, including identifying and recording intrusive memories, the trauma reminder cue procedure, delivering “mental rotation” (that is, visualizing in the mind’s eye how to rotate and move the Tetris blocks to make horizontal lines) instructions for Tetris gameplay, and collecting primary outcomes. VK participated in role-playing sessions during the workshop and subsequently with LI and MK, with filmed role-plays evaluated for protocol fidelity by an independent rater.

A written standard protocol was used by the researcher in all researcher-assisted intervention sessions to ensure intervention fidelity. Criteria for intervention fidelity were completion of an arousal level manipulation check before and after the trauma reminder cue and after Tetris gameplay; administration of the trauma reminder cue and checks to ensure the visual or perceptual details were in the participant’s mind; delivery of Tetris gameplay instructions with an emphasis on mental rotation; ensuring participants played Tetris for at least 25 minutes with at least 1 continuous period of 10 minutes. For the pilot and the first study participants, LI provided supervision and protocol fidelity checks via phone or video call after each session.

Regular supervision meetings addressed protocol fidelity and adaptations. During recruitment and data collection, VK participated in fortnightly video calls with other researchers to share experiences, best practices, and receive feedback.

#### Eligibility and Baseline Assessment Meeting: Week 1 (Preintervention)

Potential participants met with VK to assess eligibility based on the inclusion and exclusion criteria. During this meeting, the study’s purpose, procedures, risks, and benefits were explained, and verbal informed consent was obtained due to COVID-19 social distancing measures. All participants were provided with information about local occupational and mental health support services.

After giving their informed consent, participants completed the baseline assessment, including self-report measures administered via Qualtrics [42], a web-based survey platform. The “hotspots” form was then used to gather information about the number and timing of traumatic events, as well as a brief description of participants’ intrusive memories. Participants were verbally given the following definition of intrusive memories: “vivid, emotional memories of the incident that ‘pop’ into mind without warning, often taking the form of visual pictures in the mind’s eye, for example, a snapshot image or a film clip.” Participants were also informed that intrusive memories can include other senses (such as sounds and smells), that they may or may not be triggered by something the person is aware of (eg, telling someone about the incident or being back at the scene), and that intrusive memories can be “very short, fleeting, and broken up.” Participants were told that deliberately thinking about an incident, mulling it over, or having general thoughts about it without an image did not constitute an intrusive memory. Participants identified the most frequent and bothersome intrusive memory to be targeted during the intervention. Participants were instructed on how to complete a web-based daily intrusive memory diary using Qualtrics and were given clear guidelines on how to document each occurrence of an intrusive memory.

#### Intrusive Memory Monitoring and Weekly Questionnaires

Participants were asked to complete the intrusive memory diary at least once daily for 3 weeks. They received automated reminders via email and text message to ensure compliance with daily diary entries. In addition to the daily diary, participants completed weekly secondary outcome measures from week 1 (preintervention) through to week 4 (postintervention). These measures assessed various aspects of participants’ mental health and functioning (Measures and Materials section).

#### Intervention Session: Week 2 (Preintervention)

The intervention, with guided delivery by VK, targeted the most frequent and bothersome intrusive memory identified during the baseline assessment. The brief single-session imagery-competing task intervention consisted of 3 components: (1) a trauma reminder cue to bring the specific trauma memory to mind; (2) engaging in a visuospatial interference task, that is, playing the computer game Tetris for 25 minutes; and (3) using specific mental rotation instructions to play the game to optimize visuospatial demand along set timing parameters.

The trauma reminder cue consisted of participants briefly bringing the targeted intrusive memory to mind and writing down its contents (in the first person) on a blank piece of paper (similar to the method of Kessler et al [32], although with a briefer description). Participants were asked to write only as much detail as was necessary to briefly recall the memory to avoid it becoming too emotionally overwhelming. Of note, this brief trauma reminder cue is focused on specific sensory-perceptual aspects of the trauma memory, and contrary to reliving procedures included in exposure-based therapies for PTSD (eg, Shearing et al [43]), it does not include a focus on the details of the event or emotional and cognitive aspects. Furthermore, also in contrast to reliving procedures, it is brief, only just enough to bring the memory image to mind before engaging in the gameplay part of the intervention. To prevent unintended reminders, this written description was discarded by the participant immediately after the intervention session; its contents were not read by or discussed with VK, as the aim of the procedure was solely to bring the intrusive memory into working memory (ie, to activate it).

Participants then received instructions on how to play the computer game Tetris using mental rotation. To increase the visuospatial demands of the game, participants were asked to plan and work out where to place the next blocks coming up, as well as the block that was currently presented [7,24]. After receiving instructions and a demonstration from VK, participants were given the opportunity to practice the game by completing 1 or 2 lines of Tetris blocks using mental rotation before beginning the timed portion of the intervention. The intervention was delivered via the official Tetris website [44], accessed on participants’ mobile phone, or tablet computer, and the game was set to “marathon mode” and “ghost piece” off.

Participants played Tetris for at least one uninterrupted period of 10 minutes and for approximately 25 minutes in total [25]. Participants were asked to restart the game if “game over” was reached [32]. VK was present for the duration of the 25-minute gameplay to encourage participants to maintain engagement with the game and occasionally remind them about the mental rotation instructions.

#### Follow-Up: Week 4 (Postintervention)

At week 4 (postintervention), participants completed the final set of secondary outcome measures. They also completed a feedback questionnaire assessing the acceptability and feasibility of the intervention and study procedures. Participants were provided with information about the study aims and reimbursed £50 (US $65.3) for their time and effort in participating in the study.

### Measures and Materials

#### Baseline Information Regarding Traumatic Events and Intrusive Memories

Participants’ descriptions of their intrusive memories were recorded in a web-based “hotspots” form (Multimedia Appendix 2) in Qualtrics [42] using the “share screen” option.

Participants indicated how many work-related traumatic events they were currently experiencing intrusive memories of and how long ago each event took place (ie, years and months). Participants were given the following verbal instruction: “Please briefly describe the worst moments of the traumatic event(s), e.g., an image or a sound. It is fine to summarize them in just a few words.” These descriptions were then used to identify established intrusive memories, rather than only “hotspots” of trauma as in Kanstrup et al [25] and helped ensure that what participants noted were indeed intrusive memories (rather than ruminative thoughts, etc). We note that this process did not involve a detailed discussion of the traumatic event, or a focus on emotional aspects, but rather a brief description primarily focused on visuo-sensory content (ie, just a few words, such as “seeing the pattern on the cardiac monitoring machine,” as per Hoppe et al [45]). Participants were asked to label each intrusive memory by selecting a few keywords (eg, “cardiac monitoring machine”) to describe its content. These keywords were used to aid the intrusive memory diary completion as well as to identify which intrusive memory to target as part of the intervention procedure.

#### Primary Outcome Measure: Number of Intrusive Memories

Participants recorded the primary outcome, the number of intrusive memories experienced each day, in a diary, which was adapted from paper diaries used in previous studies [24,25,30,32] and here delivered using a web-based format (via Qualtrics [42]; Multimedia Appendix 3).

**Figure 2:**
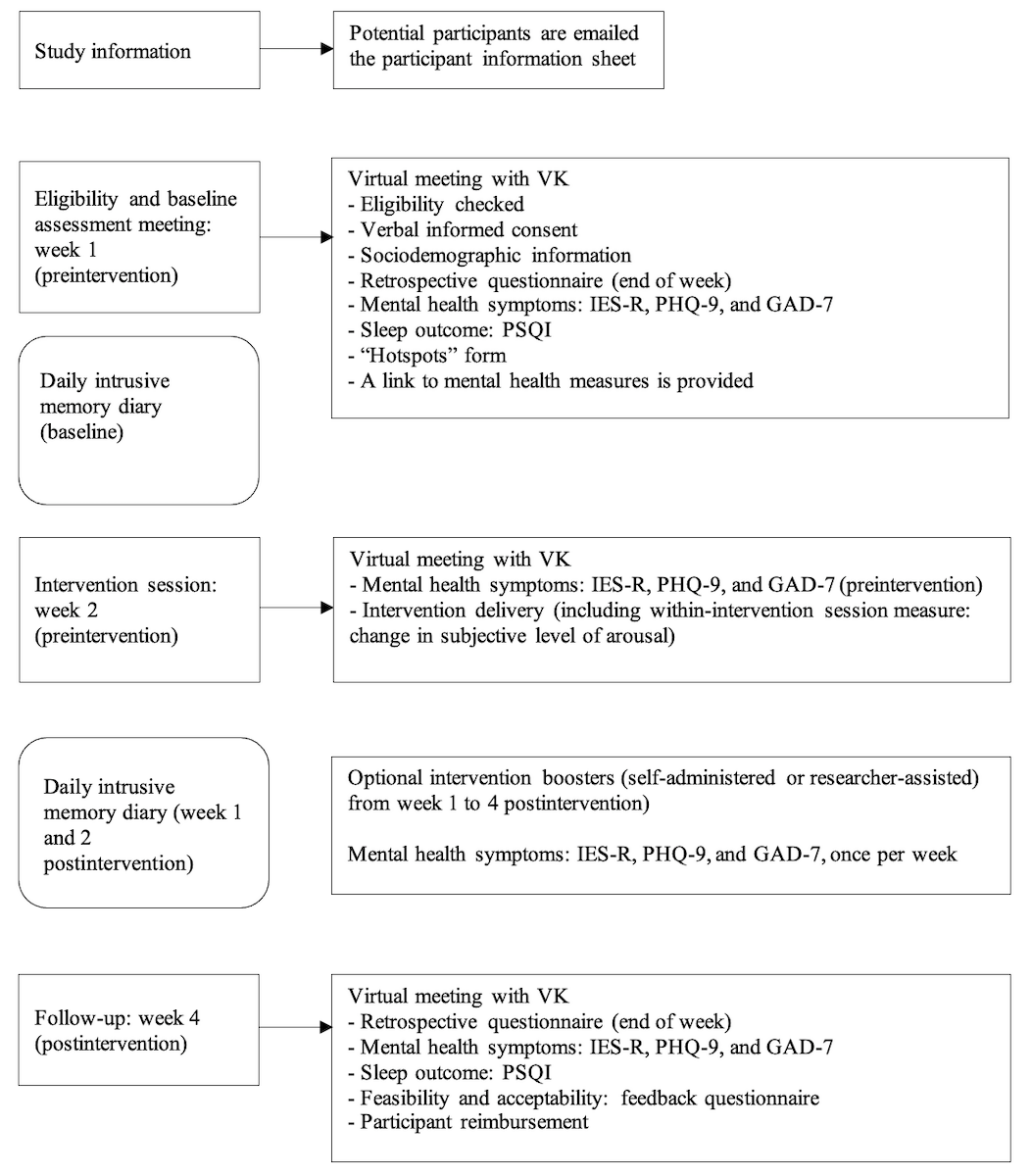
Overview of study meetings: the eligibility and baseline assessment occurred at week 1 (preintervention), followed by the intervention session at week 2 (preintervention). The final study meeting took place 4 weeks postintervention. Participants completed a daily diary for 3 weeks (1 week baseline and 2 weeks postintervention). GAD-7: Generalized Anxiety Disorder Scale; IES-R: Impact of Events Scale-Revised; PHQ-9: Patient Health Questionnaire Depression Scale; PSQI: Pittsburgh Sleep Quality Index.

Participants received daily reminders through secure email links and automated text messages to ensure diary completion. In the diary, participants were instructed to select the intrusive memories they had experienced since their last entry from a drop-down menu populated with the intrusive memories they had previously identified on the “hotspots” form. They were also asked to indicate how many times they had experienced that intrusive memory by typing in the relevant number. If they experienced a new intrusive memory (ie, not one that was identified on the “hotspots” form), they had the option to select “other.” Participants were asked to complete the diary as soon as possible after they experienced an intrusive memory, but at least once per day. If no intrusive memories were experienced that day, they were asked to indicate this by recording 0. Figure 2 depicts an overview of study meetings and when each measure was administered.

#### Secondary Outcome Measures

##### Intrusive Memory Characteristics and Impact on Functioning: Daily Intrusive Memory Diary

In the intrusive memory diary, participants rated how vivid and distressing each intrusive memory was (0=not at all to 10=extremely), how much the intrusive memory disrupted their concentration and disrupted the task at hand (0=not at all to 10=a great deal), the length of time that the intrusive memory bothered them (<1 min, 1-5 min, 6-10 min, 11-30 min, 31-60 min, and >60 min), and sleep quality over the past 24 hours (very good, fairly good, fairly bad, and very bad). Participants indicated whether they were at work when they experienced the intrusive memory, the approximate time at which this intrusive memory occurred, and how many times they used the intervention since the last diary entry (ie, to record the number of intervention booster sessions).

##### Mental Health Symptoms: Impact of Events Scale-Revised

PTSD symptoms were assessed with the Impact of Event Scale-Revised (IES-R) [46], a 22-item measure containing 3 subscales: intrusion, avoidance, and hyperarousal. Each item ranges from 0=not at all to 4=extremely. The IES-R has high internal consistency (Cronbach α=0.96) [47] and good test-retest reliability (ranging from 0.89 to 0.94) [48] and is sensitive to a general construct of traumatic stress in populations with lower symptom levels [48].

##### Mental Health Symptoms: Patient Health Questionnaire Depression Scale

Depression symptoms were assessed with the Patient Health Questionnaire-9 (PHQ-9) [49], a 9-item self-report measure of depression symptoms severity. Each item ranges from 0=not at all to 3=nearly every day. A score of ≥10 has sensitivity of 88% and specificity of 88% for major depression. The PHQ-9 has excellent internal reliability (Cronbach α=0.89) and test-retest reliability (*r*=0.84) [49], and its validity has been demonstrated in a nonclinical population [50].

##### Mental Health Symptoms: Generalized Anxiety Disorder Scale

Anxiety symptoms were assessed with the Generalized Anxiety Disorder-7 (GAD-7) [51], a brief self-report measure of symptoms of general anxiety disorder and their severity. Each item ranges from 0=not at all to 3=nearly every day. The GAD-7 has excellent internal consistency (Cronbach α=0.92) and good test-retest reliability (*r*=0.83) [51].

##### Mental Health Symptoms: Pittsburgh Sleep Quality Index

Participants rated their sleep quality with the Pittsburgh Sleep Quality Index (PSQI) [52], a self-report measure of sleep quality over a 1-month time interval. The PSQI has good internal consistency (Cronbach α=0.83) and test-retest reliability (*r*=0.85) [53].

##### Intrusive Memory Characteristics and Impact on Functioning: Retrospective Questionnaire (End of Week)

Participants rated the characteristics of their intrusive memories and their impact on functioning, retrospectively (ie, “over the past week;” Multimedia Appendix 4), using a 10-item rating scale. Participants provided ratings of the number of intrusive memories (none (0), some (1-4), quite a few (5-10), lots (11-20), very many (21-30), a large amount (31-50), and more than 50), as well as their vividness and associated distress (0=not at all to 10=extremely), the extent to which they disrupted concentration, interfered with the task at hand, affected night’s sleep, and impacted their ability to function in daily life (0=not at all to 10=a great deal). In addition, participants reported the duration of time the intrusive memories were bothersome (<1 min, 1-5 min, 6-10 min, 11-30 min, 31-60 min, and >60 min) and described how their ability to function in daily life was affected by intrusive memories.

##### Change in Subjective Level of Arousal: Within-Intervention Session Measure

Participants rated their subjective level of arousal before and after the trauma reminder cue and after playing Tetris during all researcher-assisted intervention sessions on an 11-point scale (0=calm to 10=maximum arousal). This manipulation check was done to assess changes in arousal in response to the trauma reminder cue and to check for potential immediate effects of the intervention.

##### Acceptability and Feasibility: Feedback Questionnaire

Participants completed an 11-item feedback questionnaire (adapted from Iyadurai et al [30]) to assess the acceptability of the intervention and feasibility of the study procedures (Multimedia Appendix 5). The questionnaire included items assessing participants’ experience of the intervention (ie, how easy, helpful, and burdensome they found it), their willingness to use the intervention if it was offered to them in the future, and their confidence in recommending the intervention to a colleague who was experiencing intrusive memories (0=not at all to 10=extremely). Participants also described their experience of the intervention and taking part in the study (eg, suggestions for improvements; other comments about the intervention or the study).

##### Intrusive Memory Diary Adherence

Adherence to completing the daily intrusive memory diary was assessed with the rating, “How accurately do you think you completed the diary? (0=not at all to 10=extremely).”

##### Treatment Adherence

Adherence to treatment was assessed by recording whether participants completed the trauma reminder cue procedure, received mental rotation instructions, and the duration of Tetris gameplay during the researcher-assisted intervention sessions. Adherence to mental rotation and 25-minute gameplay in self-administered sessions was not assessed.

### Data Analysis

#### Participant Characteristics

Sociodemographic data and baseline information regarding traumatic events and intrusive memories were summarized using descriptive statistics.

#### Primary Outcome Analyses

We calculated the mean number of intrusive memories per day for each participant to assess the change in the mean number of intrusive memories from baseline to postintervention. The per-day unit was chosen over per-week for greater measurement accuracy. Intrusive memories of new work-related traumatic events that occurred after participants enrolled in the study (marked as “other” in the daily intrusive memory diary) were excluded from the analyses and reported separately using descriptive statistics (Table S1 in Multimedia Appendix 1).

Baseline and postintervention daily intrusive memory means were calculated considering the exact timing of the intervention delivery within a 24-hour period. Baseline time was determined as the number of complete baseline days plus the number of hours before intervention delivery divided by 24. The baseline mean was the number of baseline intrusive memories divided by baseline time. The postintervention mean was the number of postintervention intrusive memories divided by postintervention time. The percentage change in intrusive memory frequency from baseline to postintervention was calculated as (1−[mean number per day postintervention/mean number per day at baseline]×100) [32]. A “global” percentage change in intrusive memories was calculated across all participants.

Visual inspection of individual time-series graphs is fundamental to case series methodology [54]. The time-series graphs were created using a website for single-case data analysis [55]. Visual inspection was conducted to identify patterns of change in intrusive memory frequency from baseline to postintervention [54,56].

In addition, the τ-*U* statistic was used to analyze the intervention’s impact on between-phase differences (baseline vs postintervention) [57]. Multimedia Appendix 1 gives further details regarding τ-*U* analyses.

#### Secondary Outcome Analyses

Changes in intrusive memory characteristics (ie, vividness and distress) and their impact on functioning (ie, concentration and task disruption) from baseline to postintervention were analyzed using means, tests of difference, and effect sizes. Categorical data (ie, sleep quality and length of time intrusive memories were bothersome) were presented using descriptive statistics.

We calculated means, tests of difference, and effect sizes from week 1 (preintervention) to week 4 (postintervention) to assess changes in depression, anxiety, and posttraumatic stress symptoms, global sleep, and overall sleep quality.

For retrospective ratings of intrusive memory characteristics and impact on functioning, we calculated means, SDs, and effect sizes for week 1 (preintervention) and week 4 (postintervention) ratings. Categorical data (number of intrusive memories in the past week and length of time intrusive memories were bothersome) were summarized using descriptive statistics. Qualitative data on the impact of intrusive memories on everyday functioning were presented as anonymized quotes.

Changes in subjective arousal levels (pre- to posttrauma reminder cue and pre- to post-Tetris gameplay) were reported as means and SDs, with tests of difference calculated. These findings are detailed in Multimedia Appendix 1.

Feedback questionnaire ratings were summarized using descriptive statistics to assess the intervention’s acceptability. Open-ended responses regarding the acceptability and feasibility of the intervention and study procedures were analyzed for themes, with anonymized quotes presented as examples.

Treatment adherence, including the duration of Tetris gameplay, subjective accuracy ratings for intrusive memory diary completion, and outcome measure completion rates, was summarized using descriptive statistics (Multimedia Appendix 1 provides further details).

#### Missing Data

Participants with missing data were excluded from the analysis for the specific outcome measure in which data were missing.

#### Data Accuracy Checks

Outcome data accuracy was verified by an independent rater.

### Ethical Considerations

The study required ethics approval due to the involvement of NHS staff members as participants. Ethics approval was granted by the Health Research Authority and the University of Oxford Medical Sciences Inter-Divisional Research Ethics Committee (approval number R64738/RE001). An amendment to obtain verbal consent due to COVID-19 social distancing measures was approved on April 23, 2020 (approval number R64738/RE004). Trust management approval was provided by the Oxford University Hospitals NHS Foundation Trust, the Royal Berkshire NHS Foundation Trust, and the South Central Ambulance Service NHS Foundation Trust. For public record, the study was retrospectively registered on ClinicalTrials.gov (NCT04769999) following data collection but preanalysis (Multimedia Appendix 1 provides further details).

## Results

### Intrusive Memory Diary Adherence

The mean subjective accuracy rating for daily intrusive memory diary completion across all entries and participants was 8.50 (SD 0.90; range 0-10).

### Treatment Adherence

Treatment adherence was 100% for all researcher-assisted sessions: every participant completed all 3 components of the intervention protocol (trauma reminder cue, receiving mental rotation instructions, and playing Tetris for at least 25 minutes). In these sessions, all participants played Tetris for a minimum of 25 minutes (mean 25.17, SD 0.49 min; range 25-27 min).

### Rates of Outcome Measure Completion

The completion rate for the primary outcome measure was 100%. For secondary outcome measures, the IES-R, PHQ-9, and GAD-7 completion rate was 99% (87/88; week 5 measures were missing for 1 participant). The PSQI completion rate was 96% (23/24; missing data for 1 participant). All participants completed the study feedback questionnaire.

### Attrition

All participants remained in the study for its full duration.

### Sociodemographic Information

Sociodemographic information is presented in Table 1.

**Table 1:** Participant sociodemographic characteristics (N=12).

Characteristic			Value
**Sex, n (%)**			
	Female	8 (67)	
	Male	4 (33)	
	Intersex	0 (0)	
	Prefer not to say	0 (0)	
Age (y), mean (SD; range)			32.92 (7.39; 22-49)
Education (years from first grade), mean (SD; range)			18.04 (2.75; 14-22)
**Ethnicity, n (%)**			
	White British	8 (67)	
	Any other White background	3 (25)	
	Pakistani	1 (8)	
**Relationship status, n (%)**			
	Single	7 (58)	
	Married or cohabitating	4 (33)	
	Divorced or separated	1 (8)	
**Job role, n (%)**			
	Nursing	4 (33)	
	Medical doctor	1 (8)	
	Student (medical and nursing)	2 (17)	
	Ambulance service team (eg, paramedic and emergency care assistant)	5 (42)	
**Department of employment, n (%)**			
	Emergency department	3 (25)	
	Adult intensive care unit	1 (8)	
	Neonatal intensive care unit	2 (17)	
	Other inpatient hospital ward	1 (8)	
	Ambulance service	5 (42)	
Length of time working in current job role (mo), mean (SD; range)			60.67 (71.39; 3-240)
**NHS^a^ banding level, n (%)**			
	Band 3	2 (17)	
	Band 5	4 (33)	
	Band 6	4 (33)	
	Band 7	1 (8)	
	Not applicable (medical student)	1 (8)	

^a^NHS: National Health Service.

### Baseline Information Regarding Traumatic Events and Intrusive Memories

The mean number of work-related traumatic events reported at the week 1 preintervention meeting was 2.58. Of all reported work-related traumatic events, 39% (12/31) occurred between January and September 2020, during the COVID-19 pandemic. On average, participants reported 5.33 work-related traumatic event “hotspots.” Participants reported a range of intrusive memory content; examples include patients with fatal injuries, patients dying, extremely distressed family members, and medical procedures (Table S2 in Multimedia Appendix 1 gives further details regarding baseline information).

Following the baseline period, 85 intervention sessions were delivered across all participants. Of these, 86% (73/85) were intervention boosters, with 14% (10/73) being researcher-assisted and 86% (63/73) self-administered. Table S3 in Multimedia Appendix 1 gives details of the researcher-assisted and self-administered booster sessions (ie, following the first researcher-assisted session).

### Primary Outcome Measure: Number of Intrusive Memories

Across all participants, the mean number of intrusive memories per day decreased by 59% from baseline (mean 1.29, SD 0.94) to postintervention (mean 0.54, SD 0.51). Individual-level data showed that 83% (10/12) of participants experienced reductions between 51% and 100% (Table S4 in Multimedia Appendix 1 gives individual percentage changes).

**Figure 3:**
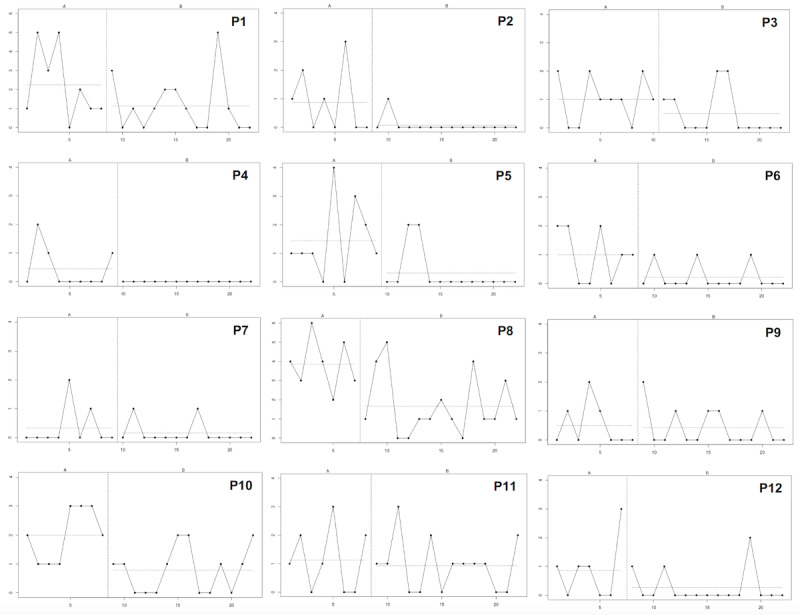
Graphs showing the primary outcome data (number of intrusive memories) for all participants (N=12). The y-axis represents the number of intrusive memories per day, and the x-axis represents each day of the study period. The horizontal dashed lines represent a measure of central tendency for the baseline (A) and postintervention (B) periods.

Visual inspection of individual time-series graphs [54,56] indicated a reduction in the number of intrusive memories following the intervention for all participants, as evidenced by overall lower measures of central tendency from baseline to postintervention (Figure 3). Generally, the number of intrusive memories decreased either immediately or soon after the initial intervention session.

Participants P2 to P5 maintained 0 intrusive memories for the final 5 consecutive days of the study. However, participants P1, P9, and P11 showed a reemergence of intrusive memories postintervention, similar to their baseline levels. Participant P1 reported several intrusive memories during a particularly stressful period at work, coinciding with the postintervention period. This period included a taxing day during the COVID-19 pandemic, including redeployment to a COVID-19 ward, associated with a sharp increase in intrusive memories on day 19. Participants P1 and P9 reported functional improvements at work and in their social lives, despite modest reductions in the number of intrusive memories.

τ-*U* analysis yielded significant (*P*<.05) medium effect sizes for 3 participants (P5, P8, and P10). The aggregated omnibus analysis showed a significant (*P*<.001) small effect size (τ-*U*=–0.38; Table S4 in Multimedia Appendix 1 gives individual participant-level data).

### Secondary Outcome Measures

#### Intrusive Memory Characteristics and Impact on Functioning: Daily Intrusive Memory Diary

A total of 216 intrusive memories were recorded by participants in the daily intrusive memory diary during the study period, with 31.9% (69/216) occurring while they were at work.

There were no statistically significant reductions in any ratings of intrusive memory characteristics (ie, vividness and distress), functioning (ie, concentration and task disruption), length of time intrusive memories were bothersome, or sleep quality, as recorded in the daily intrusive memory diary (Table S5 in Multimedia Appendix 1).

#### Mental Health Symptoms: IES-R, PHQ-9, GAD-7, and PSQI

Mental health outcomes, including depression, anxiety, and posttraumatic stress symptoms, showed significant reductions from week 1 (preintervention) to week 4 (postintervention). However, global sleep and overall sleep quality scores did not change significantly over this period (Table 2).

**Table 2:** Mental health symptoms and continuous outcomes of the retrospective ratings of intrusive memory characteristics (end of week) at week 1 (preintervention) and week 4 (postintervention), with analyses and effect sizes (N=12).

			Week 1 (preintervention), mean (SD)		Week 4 (postintervention), mean (SD)		*z* score		*t* test (*df*)		*P* value		Effect size, Cohen *d* (95% CI)
**Mental health outcome measures**													
	IES-R^a^	41.08 (15.30)		12.58 (12.72)		–3.06		—^b^		.002		—	
	PHQ-9^c^	8.83 (7.54)		3.83 (4.11)		—		3.46 (11)		.005		1.00 (0.28-1.68)	
	GAD-7^d^	7.67 (6.11)		3.58 (4.50)		–2.76		—		.006		—	
	PSQI global^e,f^	8.27 (5.10)		7.36 (5.63)		—		1.01 (10)		.34		0.31 (–0.31-0.90)	
	PSQI overall sleep quality^f,g^	1.64 (0.92)		1.18 (0.98)		—		2.19 (10)		.05		0.66 (–0.01-1.30)	
**Continuous outcomes^h^**													
	Vividness^i^	7.31 (1.75)		4.50 (1.51)		—		3.72 (7)		.007		1.32 (0.33-2.26)	
	Distress^j^	5 (1.69)		2.75 (1.58)		—		3 (7)		.02		1.06 (0.16-1.92)	
	Concentration disruption^k^	5.75 (2.38)		2.38 (1.77)		—		4.34 (7)		.003		1.53 (0.46-2.56)	
	Task disruption^l^	4 (2.39)		1.63 (1.60)		—		3.49 (7)		.01		1.24 (0.27-2.15)	
	Night’s sleep interference^m^	3.86 (3.48)		2 (3.83)		–1.84				.07		—	
	Daily functioning interference^n^	4 (2.65)		1 (1.53)		–2.23				.03		—	

^a^IES-R: Impact of Events Scale-Revised (scores ranging from 0-88).

^b^Not available.

^c^PHQ-9: Patient Health Questionnaire (scores ranging from 0-27).

^d^GAD-7: Generalized Anxiety Disorder Scale (scores ranging from 0-21).

^e^PSQI global: Pittsburgh Sleep Quality Index (global PSQI scores ranging from 0-21).

^f^n=11 (excluding P3 who had missing PSQI data for week 4 postintervention).

^g^During the past month, how would you rate your sleep quality? very good=0, fairly good=1, fairly bad=2, and very bad=3.

^h^For continuous outcomes, N=8. Data at week 1 (preintervention) have been excluded for those participants who reported no intrusive memories at week 4 (postintervention). Night’s sleep interference and daily functioning interference are missing for P1, hence N=7 for these items.

^i^How vivid were your intrusive memories? 0=not at all and 10=extremely.

^j^How distressing were your intrusive memories? 0=not at all and 10=extremely.

^k^How much did they disrupt your concentration? 0=not at all and 10=a great deal.

^l^How much did they disrupt the tasks you were doing? 0=not at all and 10=a great deal.

^m^How much did your intrusive memories interfere with your night’s sleep? 0=not at all and 10=a great deal.

^n^How much have your intrusive memories affected your ability to function in your daily life? 0=not at all and 10=a great deal.

#### Intrusive Memory Characteristics and Impact on Functioning: Retrospective Questionnaire (End of Week)

All retrospective ratings of intrusive memory characteristics showed significant reductions from week 1 (preintervention) to week 4 (postintervention), except for ratings of how much intrusive memories interfered with sleep. Participants reported fewer intrusive memories and a shorter duration of being bothered by them from week 1 (preintervention) to week 4 (postintervention; Tables 2 and 3).

**Table 3:** Discrete outcomes of the retrospective ratings of intrusive memory characteristics (end of week) at week 1 (preintervention) and week 4 (postintervention; N=12).

			Week 1 (preintervention), n (%)		Week 4 (postintervention), n (%)
**Discrete outcome^a^**					
	None	0 (0)		4 (33)	
	Some (1-4)	1 (8)		7 (58)	
	Quite a few (5-9)	4 (33)		1 (8)	
	Lots (10-20)	3 (25)		0 (0)	
	Very many (21-30)	2 (17)		0 (0)	
	A large amount (31-50)	2 (17)		0 (0)	
	More (>50)	0 (0)		0 (0)	
**Length of time that intrusive memories were bothersome^b^ (min)**					
	<1	3 (25)		4 (50)	
	1-5	5 (42)		3 (38)	
	6-10	2 (17)		1 (13)	
	11-30	1 (8)		0 (0)	
	31-60	0 (0)		0 (0)	
	>60	1 (8)		0 (0)	

^a^How many intrusive memories did you have? None (0), some (1-4), quite a few (5-10), lots (10-20), very many (21-30), a large amount (31-50), more (>50).

^b^Approximately how long did your intrusive memories bother you for? <1 minutes, 1-5 minutes, 6-10 minutes, 11-30 minutes, 31-60 minutes, and >60 minutes.

Responses to the open-ended question, “How have intrusive memories affected your ability to function in your daily life in the past week?” revealed that intrusive memories impacted various functional domains before the intervention, including occupational, cognitive, social and home life, and emotional functioning. For example, one participant stated:

I was previously reluctant to go to the next medical rotation when I have had memories about the event connected to the rotation, but now it’s felt very much in my control. It couldn’t have been a better timing to do the study.

Another participant reported, “I can focus on the tasks at hand whilst at work.” Another participant felt that they are “no longer struggling to get to sleep or waking up startled.” One participant explained, “I’m more conversive again with people; I’m more chatty with my colleagues.” Another reported:

The frustration within the family about my mentioning intrusive memories that I’ve been having...it’s not happening anymore—I’ve not been needing to mention anything to my wife at all.

One participant noted, “[My] manager noticed a difference—I’m more myself now than at the beginning of the study.” One participant observed global changes at work, “At work I’m less stressed, less moody, and happier.”

About 83% (10/12) of participants reported improved functioning at postintervention. For example, some indicated that when intrusive memories occurred, they no longer interfered with functioning. One participant noted, “The intrusive memories aren’t as vivid and they don’t last as long, so I can focus on the tasks at hand whilst at work.” Another said, “I’m still getting some of the intrusive memories, but they’re a lot less bothersome, and I feel able to manage them better.” One participant noted global changes in the impact of intrusive memories, “I don’t feel like [intrusive memories] are impacting me in any way now.”

#### Acceptability and Feasibility: Feedback Questionnaire

Participants generally found playing Tetris at work helpful and not very burdensome. There was considerable variability in participants’ ratings of how easy they found playing Tetris at work. Overall, participants found taking part in the study easy and not burdensome and reported feeling confident in suggesting playing Tetris to another staff member (Table S6 in Multimedia Appendix 1).

Participants generally found taking part in the study helpful, describing the intervention as accessible. A common theme was that playing Tetris enabled them to focus on something other than work: “It takes your concentration somewhere else.” Some found it a helpful “distraction” after an intrusive memory, “I play it from time to time following intrusive memories of other events in my life. It’s a massive distraction.”

Several participants noted a reduction in the emotional impact of their intrusive memories:

When I had the intrusive memory this week, it didn’t have such a strong emotional potency and didn’t have such a hold of me. You can deal with the memory better when it doesn’t have such a strong emotion attached to it. I can really see the benefits.

Regarding the feasibility of playing Tetris while at work, participants mentioned challenges, such as lack of time or opportunity, as for some, breaktimes were the only times they were able to access the intervention. Even then, it was sometimes perceived as antisocial:

During breaktimes is the only time that it’s possible [to play Tetris], however, even then, the staffroom is very busy and it’s hard to concentrate. You might have others asking you to join in a conversation, and you don’t want to be seen as antisocial.

Participants also highlighted potential challenges to the intervention’s acceptability in their work environments, such as: “I didn’t feel comfortable telling my nurse in charge that I need to go play Tetris,” and:

When colleagues describe intrusive memories, the expectation is that we sit there and listen. Culturally, we are not there yet to do something that might distract away from the memory. It might come across insensitive or that I’m not interested.

Finally, participants recommended improvements, such as having Tetris on an app, a version without advertisements, shorter play sessions, and regular reminders to play Tetris.

## Discussion

### Principal Findings

This single-case series examined the efficacy, feasibility, and acceptability of a brief imagery-competing task intervention to reduce intrusive memories in frontline NHS health care staff, including both prehospital and hospital staff, exposed to workplace trauma. Overall, participants reported a substantial reduction (59%) in the number of intrusive memories per day from baseline to postintervention. All participants experienced a decrease in daily intrusive memories from baseline to postintervention, with reductions ranging from 51% to 100% in 10 of 12 (83%) participants. In addition, there was a notable improvement in depression, anxiety, and posttraumatic stress symptoms over the intervention period, although no change in sleep patterns was observed. Qualitative feedback indicated enhanced cognitive, behavioral, and emotional functioning across social, home life, and work-related domains following the intervention, emphasizing its feasibility and acceptability. Adherence to treatment protocols was excellent.

This study supports previous findings that indicate the effect of the intervention in reducing intrusive memories among trauma-exposed populations (eg, inpatients with complex PTSD [32], refugees [31], and ICU staff [36,37]), extending to various frontline health care staff employed in prehospital contexts, such as ambulance services, as well as hospital staff (eg, in the ED and ICU). Alongside reduced intrusive memory frequency, improvements in psychopathology symptoms (eg, posttraumatic stress symptoms) and overall functioning were observed, in accordance with evidence that intrusive symptoms are centrally linked to other PTSD symptoms [10].

Participants also reported significant reductions in intrusive memory–related distress and vividness, along with positive changes in their appraisals of intrusive memories postintervention, as assessed by the retrospective questionnaire (end of week). For example, following the intervention, participants rated their intrusive memories as less “bothersome,” and in qualitative responses, some reported feeling better able to manage intrusive memories when they did come to mind. However, significant changes were not observed when the characteristics of intrusive memories were measured in the daily intrusive memory diary. The discrepancies between the daily and retrospective measures of intrusive memory characteristics may be due to differences in data collection methods. Daily diary entries capture immediate, specific experiences, while retrospective ratings reflect a broader, overall assessment of intrusive memories over the past week, potentially encompassing global changes in participants’ perceptions and impact on functioning. Thus, although the primary objective of the intervention is to reduce the number of intrusive memories, our findings suggest possible broader clinical impact. Future evaluations should consider measuring outcomes beyond the frequency of intrusive memories, such as their qualities, associated appraisals, symptoms of psychopathology, and functional impacts, to capture the broader potential effects of the intervention.

The COVID-19 pandemic had an unavoidable impact on this research, necessitating several modifications to the study design and procedures after the study had commenced. First, the study duration was reduced, and it was not possible to proceed with the originally planned randomization to a 3- or 5-week baseline, which we acknowledge as a limitation. Second, due to the national lockdown, study procedures were adapted for remote rather than in-person delivery. Nevertheless, delivering the intervention according to the protocol despite these modifications underscores its flexibility and potential for remote delivery—2 key features of a scalable intervention.

### Study Limitations

We acknowledge some important limitations of the study that should be considered when interpreting the findings. First, we used an AB single-case experimental design. Further research has since included randomized procedures to test the effectiveness and feasibility of the intervention for NHS staff, as demonstrated by Ramineni et al [36] and Iyadurai et al [37] for ICU staff. In addition, future studies could benefit from incorporating additional time-related controls, such as the stability of daily intrusive memories over time, the typical frequency of intrusive memories in the week before the study, and the long-term effects of the intervention. Finally, this study design does not allow us to distinguish the effects of specific intervention components, such as discarding the written descriptions of the intrusive memories. Further research with varied designs is needed to isolate and understand the impact of each individual component of the procedure.

### Conclusions

In conclusion, this case series supports the efficacy of an imagery-competing task intervention in reducing the number of intrusive memories and improving mental health and functioning among frontline NHS prehospital and hospital staff, aligning with findings in other populations. Important next steps have included further optimizing the digital delivery of the intervention across various NHS health care staff groups and settings, using rigorous randomized controlled designs (eg, with an active control), and investigating longer-term effects of the intervention [36,37]. These and similar future trials will further enhance the development of a remotely delivered, scalable intervention aimed at mitigating the impact of work-related traumatic events on health care staff globally.

## Appendix

Multimedia Appendix 1 Data pertaining to pilot participants 1 and 2, further information for primary and secondary outcome analyses, further information regarding ethics approval, results from the within-intervention session measure, and supplementary tables.

Multimedia Appendix 2 “Hotspots” form.

Multimedia Appendix 3 Intrusive memory diary.

Multimedia Appendix 4 Retrospective questionnaire (end of week).

Multimedia Appendix 5 Feedback questionnaire.

## Abbreviations

CONSORT: Consolidated Standards of Reporting Trials
ED: emergency department
GAD-7: Generalized Anxiety Disorder-7
ICU: intensive care unit
IES-R: Impact of Event Scale-Revised
NHS: National Health Service
PHQ-9: Patient Health Questionnaire-9
PSQI: Pittsburgh Sleep Quality Index
PTSD: posttraumatic stress disorder
