# Supplementary material for: Reducing the Number of Intrusive Memories of Work-Related Traumatic Events in Frontline Health Care Staff During the COVID-19 Pandemic: Case Series
Source: JMIR Hum Factors. 2024 Nov 18;11:e55562. doi: 10.2196/55562 (PMC11612583; doi:10.2196/55562)
Supplement: Multimedia Appendix 1 [file humanfactors_v11i1e55562_app1.pdf]

Supplementary Materials

**Reducing the Number of Intrusive Memories of Work-Related Traumatic Events in Frontline Healthcare Staff:
A Case Series during the COVID-19 Pandemic**

Veronika Kubickova, DClinPsy; Craig Steel, PhD; Michelle L. Moulds, PhD; Marie Kanstrup, PhD; Sally Beer, PG Cert; Melanie Darwent, BM BCh; Liza Keating, MB ChB, MSc; Emily A. Holmes, PhD; Lalitha Iyadurai, PhD

Contents

Ethical approval: ClinicalTrials.gov registration (NCT04769999) 2

Pilot participants 1 and 2 2

Further information for primary outcome analyses 12

Further information for secondary outcome analyses: change in subjective level of arousal (within-intervention session measure) 13

Further information for secondary outcome analyses: treatment adherence 13

Supplementary Tables 14

- Supplementary Materials Table of new intrusive memories 14
- Supplementary Materials Table of baseline information 14
- Supplementary Materials Table of intervention boosters 14
- Supplementary Materials Table of Tau-U analysis and mean percentage reductions 15
- Supplementary Materials Table of intrusive memory characteristics and impact on functioning: daily intrusive memory diary 16
- Supplementary Materials Table of acceptability and feasibility: feedback questionnaire 17

References 18

Ethical approval: ClinicalTrials.gov registration (NCT04769999)

ClinicalTrials.gov registration (NCT04769999) states "The baseline period and the postintervention period were defined separately for each intrusive memory, according to when that intrusion was first targeted with the intervention", i.e., each intrusive memory had a different baseline and postintervention period, depending on when it was first targeted with the intervention. In this report we defined and analysed the baseline period as the monitoring-only phase and the postintervention period as the time after the intervention was first administered, in line with advice received at the end of the study (during VK's DClinPsy Viva) from experts in case series methodology.

Pilot participants 1 and 2

Methods

The Methods described here only highlight the differences in those used for the pilot participants and the main study participants, P1 to P12. Please refer to the main manuscript for a full description of Methods.

Participants

Pilot 1 and 2 were recruited in February 2020, before COVID-19 national restrictions, including social distancing, were enforced. Due to study design changes outlined below, Pilot 1 and Pilot 2 inclusion and exclusion criteria differed slightly to those used for P1 to P12. Inclusion and exclusion criteria for pilot participants are given below.

Pilot 1:

Inclusion criteria

- Aged 18 or above
- Able to read, write and speak in English

- Able and willing to provide informed consent and complete study procedures
- Clinical staff working in urgent care hospital departments at the John Radcliffe and Royal Berkshire Hospital
- Experiencing intrusive memories, which are problematic to them (self-report)
- Reporting the occurrence of at least two intrusive memories of a work-related traumatic incident in the week prior to beginning the study
- Able and willing to talk about the intrusive memories
- Able and willing to complete an electronic intrusive memory diary over a six-week period
- Able and willing to play Tetris on a hand-held device
- Not currently undergoing treatment for PTSD or its symptoms

Exclusion criteria

- A participant will be excluded if they have less than or equal to 1 targeted intrusive memory per week for all weeks during the baseline period.
- Participants will also be excluded if they start undergoing treatment for PTSD or its symptoms during the course of the study.

Pilot 2:

Inclusion criteria

- Aged 18 or above
- Able to read, write and speak in English
- Able and willing to provide informed consent and complete study procedures
- Clinical staff working in urgent care hospital departments at the John Radcliffe and Royal Berkshire Hospital

- Experiencing intrusive memories, which are problematic to them (self-report)
- Reporting the occurrence of at least two intrusive memories of a work-related traumatic incident in the week prior to beginning the study
- Able and willing to talk about the intrusive memories
- Able and willing to complete an electronic intrusive memory diary over an eight-week period
- Able and willing to play Tetris on a hand-held device
- Not currently undergoing treatment for PTSD or its symptoms

Exclusion criteria

- A participant will be excluded if they have less than or equal to 1 targeted intrusive memory per week for all weeks during the baseline period.
- Participants will also be excluded if they start undergoing treatment for PTSD or its symptoms during the course of the study.

Design

The initial design was a between-subjects multiple baseline AB design [1], in which participants were randomised to a 3- or 5-week baseline period. Following initial difficulties with recruitment and national restrictions put in place owing to the COVID-19 pandemic, the overall duration of the study period was reduced, and randomisation was not implemented after Pilot 2, to improve recruitment opportunities and minimise burden on frontline healthcare staff. The study design was amended three times. As a result, Pilot 1 completed a six-week study design (three-week baseline period and three-week postintervention period following the administration of a single researcher-assisted intervention session), Pilot 2 completed an eight-week study design (three-week baseline period and a five-week

postintervention period. Pilot 2 completed three separate researcher-assisted interventions targeting three intrusive memories; see Figure 1).

Figure 1. Overview of study meetings: Eligibility and baseline assessment occurred at week 1 (preintervention), followed by the intervention session at week 2 (preintervention). The final study meeting took place five weeks postintervention for Pilot 1, and seven weeks postintervention for Pilot 2. Pilot 1 completed a daily intrusive memory diary for six weeks (three weeks baseline and three weeks postintervention), and Pilot 2 completed the daily intrusive memory diary for eight weeks (three weeks baseline and five weeks postintervention). *Note*: the intervention sessions were conducted face-to-face for Pilot 1 and remotely via videocall for Pilot 2 due to COVID-19 restrictions.

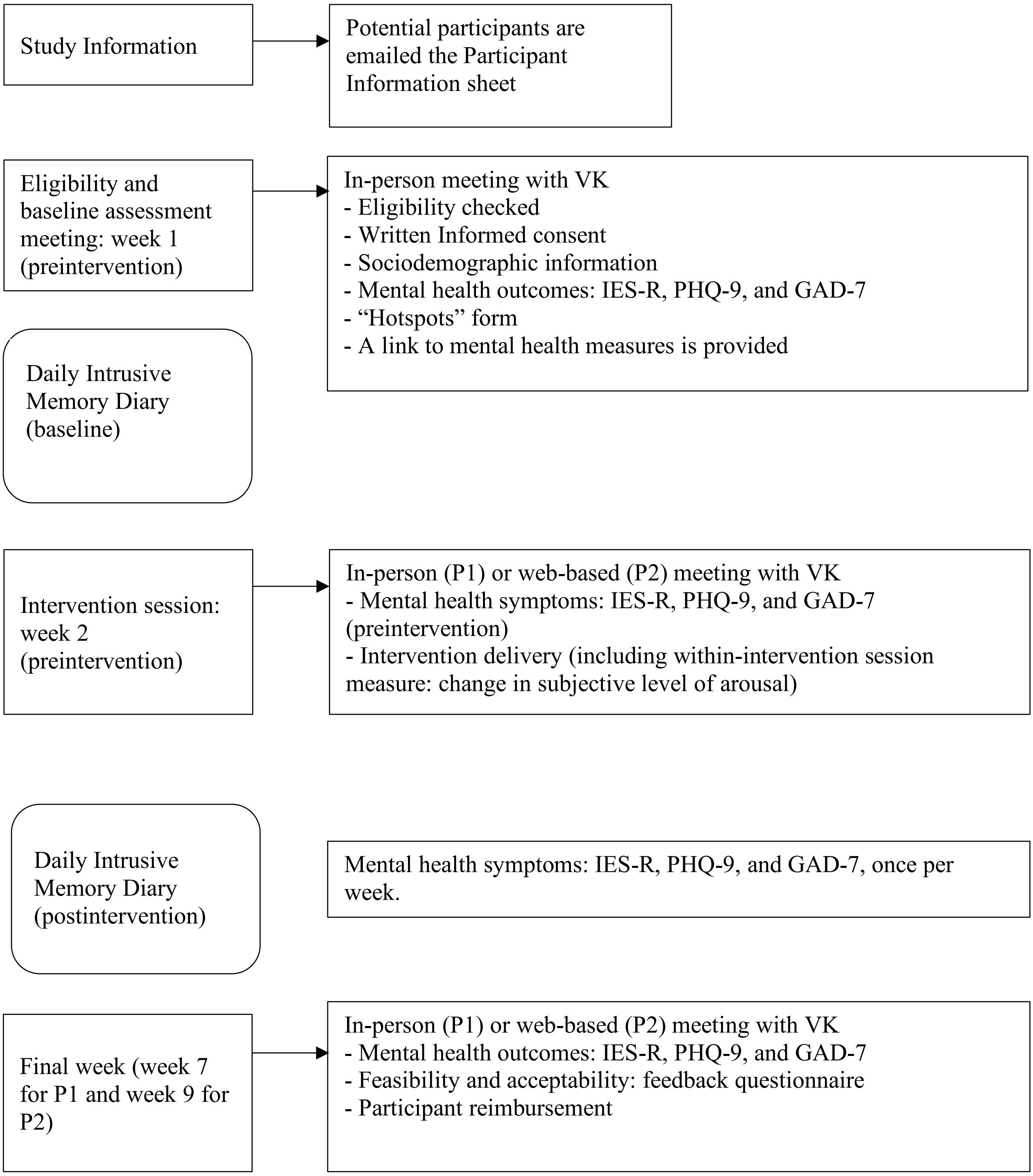

Intervention session

Pilot 1 played Tetris on a Nintendo DS XL. Due to the COVID-19 pandemic resulting in national lockdown measures, the intervention was delivered remotely to Pilot 2, via the official Tetris website [2] accessed on their mobile phone or tablet computer.

Data analysis

The primary outcome analyses are the same for Pilot 1 and 2 as they are for P1-P12 and are described in the main manuscript. The secondary outcome analyses were summarised using descriptive statistics.

Results

Treatment adherence was 100% for all researcher-assisted sessions: both pilot participants completed all three components of the intervention protocol (trauma reminder cue procedure, receiving mental rotation instructions, and playing Tetris for at least 25 minutes). Both pilot participants played Tetris for 25 minutes during all researcher-assisted intervention session.

Rates of outcome measure completion and attrition

Outcome measure completion

Two days of intrusive memory diary data were missing for Pilot 1 and two days for Pilot 2 (4/362; 0.6%). All secondary outcome measures were completed by Pilot 1 and Pilot 2.
The mean subjective accuracy rating for intrusive memory diary completion across all entries for Pilot 1 was 9.38 (SD 0.98; range 7-10) and for Pilot 2 was 5.97 (SD 1.77; range 0-10).

Attrition

Both pilot participants remained in the study for its full duration.

Sociodemographic information

Limited sociodemographic information is provided for Pilot 1 and 2, to preserve their confidentiality. Pilot 1 and 2 were both white British nurses (male, n=1; female, n=1) working in the emergency departments.

Baseline information regarding traumatic events and intrusive memories

Pilot 1 reported three distinct traumatic events with three 'hotspots'. The traumatic events took place between three and six years prior to recruitment. Pilot 2 two reported two distinct traumatic events with four 'hotspots', with the events taking place between one and two years prior to recruitment. Pilot 1 and 2 reported intrusive memory content related to patients dying, serious physical injuries, and the impact of work-related trauma on colleagues.

A total of 12 intrusive memories were recorded by Pilot 1 in the intrusive memory diary during the six-week study period with, 8% occurring while they were at work. For Pilot 2, 55% of their 128 intrusive memories recorded in the intrusive memory diary during the eight-week study period, occurred at work.

Primary outcome measure: *number of intrusive memories*

The mean number of intrusive memories per day decreased by 79% from baseline (mean 0.49) to postintervention (mean 0.10) for Pilot 1, and by 47% from baseline (mean 3.19) to postintervention (mean 1.69) for Pilot 2.

For Pilot 1, the Tau-*U* analysis yielded a Tau value of -0.31 (SD 0.31; 90% CI -0.61 to -0.02). This suggests a negative trend, although the result was not statistically significant (*P*=.08). For Pilot 2 participants, with baseline-corrected data, the Tau-*U* analysis showed a statistically significant (*P*=.003) Tau value of -0.48 (SD 0.16; 90% CI -0.74 to -0.22). This indicates a significant and robust medium negative effect.

Visual inspection of individual time-series graphs [3,4] indicated a reduction in the mean number of intrusive memories following the intervention for both Pilot participants, as evidenced by the decreasing measures of central tendency from baseline to postintervention (see Figure 2). The number of intrusive memories decreased immediately for Pilot 1 after the intervention session. For Pilot 2, there was a delayed response, where there was an increase in the number of intrusive memories in the day after the intervention. However, the overall trend

was one of a gradual reduction in the number of intrusive memories over the postintervention period.

Compared to Pilot 1, for Pilot 2, the graph reveals a more pronounced and consistent downward trend. The reduction in the number of intrusive memories is more evident and stable in Pilot 2, aligning with the statistically significant negative effect observed in the Tau-*U* analysis. These visual patterns corroborate the quantitative findings, highlighting the stronger and more reliable negative trend observed for Pilot 2.

Figure 2. Graphs showing the primary outcome data (number of intrusive memories) for Pilot participants 1 and 2. The y-axis represents the number of intrusive memories per day, and the x-axis represents each day of the study period. The horizontal dashed lines represent the measure of central tendency for the baseline (A) and postintervention (B) periods.

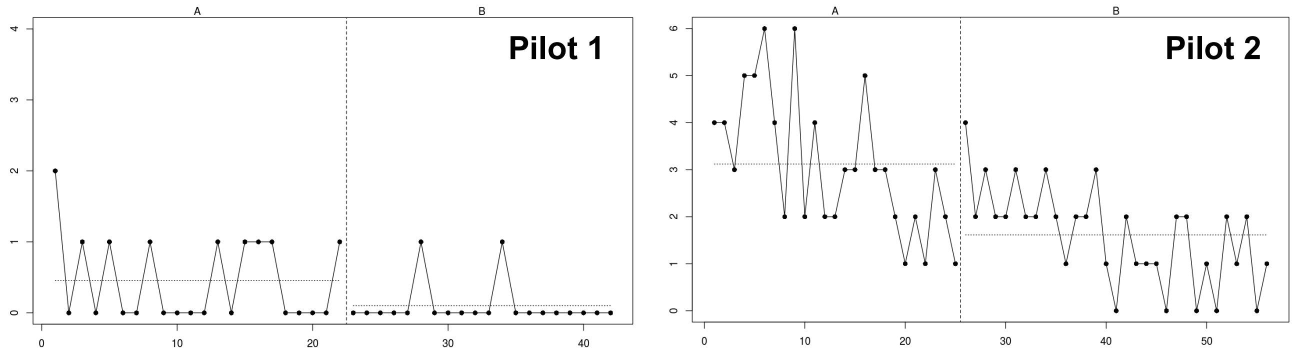

Secondary outcome measures

Intrusive memory characteristics and impact on functioning: intrusive memory diary

There was a decrease in ratings of intrusive memory distress and vividness, from baseline to postintervention for both participants. While disruption to concentration and task at hand decreased for Pilot 2, there was a slight increase in ratings for Pilot 1, from baseline to postintervention (see Table 1).

Table 1. Ratings of intrusive memory characteristics and impact on functioning at baseline ('A') and postintervention ('B') as recorded in the daily intrusive memory diary by Pilot 1 and 2.

	Distress		Vividness		Concentration		Task disruption	
	'A' mean (SD)	'B' mean (SD)	'A' mean (SD)	'B' mean (SD)	'A' mean (SD)	'B' mean (SD)	'A' mean (SD)	'B' mean (SD)
Pilot 1	6.4 (1.17)	5.0 (4.24)	6.8 (1.62)	6 (1.41)	3.2 (2.10)	3.5 (2.12)	1.9 (1.37)	3 (2.83)
Pilot 2	5.22 (1.90)	5.06 (2.00)	6.26 (1.82)	5.2 (2.12)	4.33 (2.14)	3.04 (1.43)	3.69 (2.28)	2.24 (1.24)

Mental health symptoms: IES-R, PHQ-9, and GAD-7

All mental health outcomes reduced from week 1 (preintervention) to week 7 (postintervention) for Pilot 1, and from week 1 (preintervention) to week 9 (postintervention), for Pilot 2 (see Table 2).

Table 2. Changes in mental health symptoms: IES-R, PHQ-9, and GAD-7, from week 1 (preintervention) to week 7 (post0intervention) for Pilot 1, and from week 1 (preintervention) to week 9 (postintervention), for Pilot 2.

	IES-R		PHQ-9		GAD-7	
	Preintervention	Postintervention	Preintervention	Postintervention	Preintervention	Postintervention
Pilot 1	30	7	15	3	9	4
Pilot 2	49	16	15	3	10	2

Acceptability and feasibility: feedback questionnaire

Pilot 1 and 2 generally found playing Tetris at work helpful, easy, and not very burdensome. Pilot 1 indicated that they found taking part in the study somewhat burdensome, whereas Pilot 2 did not find the study burdensome. All responses on the Feedback questionnaire are given in Table 3. Pilot 2 answered one qualitative question regarding recommendations to improve the study. They stated that they would find it helpful to have a later reminder to complete the daily intrusive memory diary when they are working night shifts. No other qualitative questions were answered by either participant.

Table 3. Pilot 1 and 2 responses on Likert scale items Feedback questionnaire.

	Pilot 1	Pilot 2
How easy did you find playing Tetris whilst at work?[a]	9	9
How helpful did you find playing Tetris whilst at work?[b]	9	9
How burdensome did you find playing Tetris whilst at work?[c]	1	0
If you were experiencing intrusive memories in the future, how willing would you be to play Tetris if it was offered to you as something that would help?[d]	10	8
If another staff member was having intrusive memories, how confident would you be in suggesting playing Tetris to them?[e]	7	9
How easy did you find taking part in the study?[f]	7	9
How burdensome did you find taking part in the study?[g]	7	1

[a]0=not at all easy, to 10=extremely easy
[b]0=not at all helpful, to 10=extremely helpful
[c]0=not at all burdensome, to 10=extremely burdensome
[d]0=extremely unwilling, to 10=extremely willing
[e]0=extremely unconfident, to 10=extremely confident
[f]0=not at all easy, to 10=extremely easy
[g]0=not at all burdensome, to 10=extremely burdensome

Change in subjective level of arousal: within-intervention session measure

All researcher-assisted intervention session for Pilot 1 and 2, apart from one for Pilot 2, showed an increase in subjective arousal ratings from pre- to post-trauma reminder cue, and then a decrease following Tetris gameplay; this pattern likely indicates successful memory reactivation using the trauma reminder cue procedure (see Table 4).

Table 4. Changes in subjective levels of arousal for Pilot 1 and 2 for all researcher-assisted intervention sessions.

	Pre-memory reminder	Post-memory reminder	Post-Tetris
Pilot 1	3	8	4
Pilot 2	3	8	4
	9	9	6
	4	5	3

Lessons learned from Pilot participants

Learning from the pilot participant data contributed to several decisions that were adopted for the main study participants. Firstly, a shorter study design (1-week preintervention and 2-weeks postintervention) was implemented after exploring the reasons for poor initial uptake into the study (e.g., busy clinical staff) with the study collaborators (e.g., emergency department research nurses). It was predicted that shortening the study period would make the study less burdensome for already busy clinical staff and improve recruitment rates.

Secondly, we expended recruitment to include pre-hospital staff, (e.g., paramedics) from South Central Ambulance Service (SCAS) NHS Foundation Trust.

Third, a decision was made to include a follow-up period four weeks after the delivery of the intervention for the main study participants to assess the impact of the intervention in the long-term.

Next, a subjective assessment of sleep quality ("Over the past 24 hours, how would you rate your sleep quality?") was added to the daily intrusive memory diary given the emerging literature regarding a significant relationship between intrusive memories and sleep [5].

Further, an additional measure, a 10-item rating scale of retrospective ratings of intrusive memory characteristics was included at the week 1 (preintervention) meeting and at the week 4 (postintervention meeting) for all study participants. The rationale for this was to assess, over the past week, participants' experience of intrusive memories over the past week

and, in doing so, capture their perceptions of how intrusive memories impact various aspects of their functioning overall.

Finally, both researcher-assisted and self-administered intervention boosters were implemented to support continuous targeting of intrusive memories if they recurred after the initial researcher-assisted intervention session.

Ethical amendments (R64738-RE003; R64738-RE004; R64783-RE005) were submitted and approved prior to commencing the above-named study changes.

Further information for primary outcome analyses

Tau-*U* statistic was used as a subsidiary analysis to explore intervention effects on the mean number of intrusive memories. Individual participant data was examined for evidence of a baseline trend using a web-based calculator [6]. Following inputting the baseline data into the calculator, the calculator makes a recommendation as to whether to estimate an effect size using an uncorrected Tau analysis, or indeed, whether a baseline-corrected Tau is required. When a baseline trend is found, this indicates that significant changes between data pairs are occurring in the absence of an intervention effect. If a significant change in the trend within the baseline was detected, effect sizes were estimated from the baseline trend-corrected data [6]. Tau-*U* was subsequently calculated using the web-based Tau-*U* calculator [7]. Individual phase contrasts between the baseline and the adjacent postintervention contrast were calculated for each participant. Results were then aggregated into a single omnibus effect size for the series [4]. Tau-*U* effect sizes are measured as *small* (0-0.65), *medium* (0.66-0.92), or *large* (0.93-1.00) [8]. New intrusive memories which were not reported at baseline assessment, i.e., prior to baseline monitoring commencing, were excluded from the main analysis and were reported descriptively for both baseline and postintervention periods.

Further information for secondary outcome analyses: change in subjective level of arousal (within-intervention session measure)

All participants reported an increase in subjective arousal ratings from pre- to post-memory reminder procedure, and then a decrease following Tetris gameplay; this pattern likely indicates successful memory reactivation using the memory reminder procedure. Specifically, mean ratings across each of the 23 intrusive memories targeted within researcher-assisted intervention sessions increased from 2.35 (SD 1.23) at the pre-memory reminder stage to 5.78 (SD 1.13) immediately following the memory reminder procedure. This change was statistically significant, Z=-4.23, P<.001. The mean ratings subsequently decreased to a mean of 2.00 (SD 1.31) following Tetris gameplay, representing a statistically significant change, Z=-4.23, P<.001.

Further information for secondary outcome analyses: treatment adherence

With regards to rates of outcome measure completion, for the primary outcome, this was calculated as the total number of days on which the diary was completed for all participants divided by the total number of days for which the diary should have been completed for all participants multiplied by 100. For secondary outcome measures, this was calculated as the total number of weeks on which each weekly questionnaire was completed for all participants out of the total possible number of weeks on which each weekly questionnaire should have been completed for all participants, multiplied by 100.

Mean duration of Tetris gameplay was calculated across all researcher-assisted intervention sessions for all participants.

Supplementary Tables

Supplementary Materials Table of new intrusive memories

Table S1. New intrusive memories of work-related traumatic events recorded by participants (N=3). *Note*: new intrusive memories were identified by participants by selecting ‘Other’ from the drop-down menu in the daily intrusive memory diary.

Participant	Total number of intrusive memories excluding ‘Other’	Number of different ‘Other’ intrusive memories	Number of ‘Other’ intrusive memories at baseline	Number of ‘Other’ intrusive memories at postintervention
P9	10	1	0	1
P10	27	5	4	2
P12	10	1	1	1

Supplementary Materials Table of baseline information

Table S2. Details of work-related traumatic events reported at week 1 (preintervention) meeting for all participants (N=12).

	n (%)	Mean (SD)	Range
Number of discrete traumatic events	31 (100%)	2.58 (1.98)	1 – 8
Year discrete traumatic events occurred			
Jan 2020 – Sept 2020 (during COVID-19 pandemic)	12 (39%)		
2019 – 2016	14 (45%)		
2015 – 2005	5 (16%)		
Number of different ‘hotspots’ of work-related traumatic events		5.33 (3.06)	2 – 14

Supplementary Materials Table of intervention boosters

Table S3. Intervention booster sessions (i.e., intervention sessions after the first researcher-administered session). Number of researcher-assisted and self-administered booster sessions per participant.

Participant	Total number of booster sessions administered	Number of researcher-assisted booster sessions	Number of self-administered booster sessions
P1	4	0	4
P2	1	0	1
P3	4	0	4
P4	0	N/A[a]	N/A
P5	3	1	2
P6	3	0	3
P7	2	2	0
P8	23	1	22
P9	7	3	4
P10	10	1	9
P11	13	0	13
P12	3	2	1

[a]Not applicable.

Supplementary Materials Table of Tau-*U* analysis and mean percentage reductions

Table S4. Tau-*U* analysis and mean percentage reductions. This table displays the effect of the intervention for each participant (N=12), comparing individual baseline and postintervention data. It also includes the aggregated omnibus effect size. Individual mean number of intrusive memories and percentage reductions per day from baseline to postintervention are also shown.

Participant	Tau (SD)	*P*	90% CI	Mean no. of intrusive memories at baseline	Mean no. of intrusive memories at postintervention	% reduction
P1	-0.38 (0.26)	0.142	-0.81, 0.05	2.32	1.12	51%
P2	-0.45 (0.26)	0.090	-0.88, -0.02	0.91	0.07	92%
P3	-0.35 (0.25)	0.166	-0.77, 0.07	1.02	0.49	52%
P4	-0.33 (0.26)	0.193	-0.75, 0.09	0.46	0.00	100%
P5	-0.59 (0.26)	0.021[a]	-1.00, -0.17	1.46	0.31	79%
P6	-0.49 (0.26)	0.061	-0.92, -0.06	1.03	0.21	79%
P7	-0.13 (0.26)	0.633	-0.56, 0.31	0.31	0.16	47%
P8	-0.70 (0.27)	0.010[a]	-1.00, -0.25	3.54	1.74	51%
P9	-0.04 (0.26)	0.891	-0.47, 0.40	0.53	0.41	22%
P10	-0.65 (0.26)	0.013[a]	-1.00, -0.22	2.11	0.76	64%
P11	-0.09 (0.26)	0.733	-0.52, 0.34	1.06	0.96	10%
P12	-0.37 (0.27)	0.169	-0.82, 0.07	0.79	0.28	65%

Total			59%
Aggregated omnibus analysis	Tau (variance)		95% CI
Weighted average	-0.38 (0.08)	<.001	-0.53, -0.23

[a]Statistically significant AB comparisons at *P*< .05 level.

Supplementary Materials Table of intrusive memory characteristics and impact on functioning: daily intrusive memory diary

There were no statistically significant reductions in the ratings of intrusive memory vividness from baseline (mean 6.20, SD 1.97) to postintervention (mean 5.89, SD 1.71), $t(10)=.86$, $P=.41$; in the ratings of distress from baseline (mean 4.90, SD 1.70) to postintervention (mean 5.12, SD 1.78), $t(10)=-.84$, $P=.42$; in the ratings of disruption to concentration from baseline (mean 4.30, SD 2.25) to postintervention (mean 4.61, SD 2.17), $t(10)=-.72$, $P=.49$; or in the ratings of disruption to tasks from baseline (mean 3.82, SD 1.93) to postintervention (mean 3.79, SD 1.89), $t(10)=.11$, $P=.91$ (see Table S5 below).

Additionally, there was no meaningful reduction in the length of time that intrusive memories were bothersome from baseline to postintervention, nor was there a significant change in sleep quality from baseline to postintervention.

Table S5. Mean and standard deviations for intrusive memory characteristics and impact on functioning at baseline (A) and postintervention (B) per participant (N=12).

	Distress		Vividness		Concentration		Disrupt task	
Participant	'A' mean (SD)	'B' mean (SD)	'A' mean (SD)	'B' mean (SD)	'A' mean (SD)	'B' mean (SD)	'A' mean (SD)	'B' mean (SD)
P1	1.83 (2.07)	3.31 (2.33)	4.44 (1.29)	6.19 (2.14)	1.33 (1.78)	4.89 (2.70)	3.83 (4.08)	4.81 (3.53)
P2	3.71 (0.76)	3.00	3.71 (0.95)	5.00	3.14 (1.68)	4.00	2.00 (1.15)	4.00
P3	4.30 (1.06)	4.00 (0.89)	7.90 (1.97)	7.17 (1.83)	3.4 (0.97)	3.33 (1.21)	2.70 (0.67)	2.67 (0.52)
P4	4.00 (2.16)	N/A[a]	4.50 (1.73)	N/A	3.00 (1.83)	N/A	2.50 (1.29)	N/A

P5	6.08 (1.04)	6.50 (1.00)	9.69 (0.85)	8.00 (0)	3.92 (1.12)	4.00 (0.82)	3.62 (1.12)	3.00 (0.82)
P6	2.63 (1.77)	2.67 (0.58)	3.75 (2.25)	3.67 (1.15)	0.00	0.00	0.13 (0.35)	0.00
P7	4.67 (0.58)	6.50 (0.71)	6.33 (0.58)	7.00 (0)	6.67 (0.58)	8.00 (2.83)	5.67 (2.52)	4.5 (3.54)
P8	6.59 (1.34)	6.88 (0.88)	7.44 (1.45)	6.44 (2.12)	6.15 (1.23)	6.36 (1.04)	5.93 (1.64)	6.28 (1.57)
P9	4.75 (1.26)	3.83 (0.75)	4.00 (1.41)	2.33 (0.52)	4.25 (1.26)	3.33 (1.21)	3.25 (0.96)	3.17 (1.17)
P10	6.13 (1.26)	5.55 (1.04)	7.38 (1.09)	6.91 (1.04)	6.38 (1.54)	4.09 (1.30)	3.75 (2.29)	2.36 (1.21)
P11	7.33 (2.24)	7.54 (1.81)	6.89 (2.09)	7.08 (1.55)	6.89 (2.03)	7.00 (1.58)	7.11 (2.32)	6.85 (1.68)
P12	5.83 (1.72)	6.50 (0.58)	6.67 (1.51)	5.00 (2.58)	5.17 (1.72)	5.75 (0.50)	4.00 (2.10)	4.00 (0.82)

[a]Not applicable. The participant did not report any intrusive memories during that period.

Supplementary Materials Table of acceptability and feasibility: feedback questionnaire

Table S6. Feedback questionnaire means, standard deviations and ranges for responses on Likert scale items (N=12).

	Mean (SD)	Range
How easy did you find playing Tetris whilst at work?[a]	3.83 (2.52)	0 – 9
How helpful did you find playing Tetris whilst at work?[b,c]	7.00 (2.45)	2 – 10
How burdensome did you find playing Tetris whilst at work?[c,d]	3.00 (2.45)	0 – 7
If you were experiencing intrusive memories in the future, how willing would you be to play Tetris if it was offered to you as something that would help?[e]	8.92 (1.00)	8 – 10
If another staff member was having intrusive memories, how confident would you be in suggesting playing Tetris to them?[f]	7.50 (1.73)	5 – 10
How easy did you find taking part in the study?[g]	9.08 (1.16)	6 – 10
How burdensome did you find taking part in the study?[h]	1.67 (1.30)	0 – 7

[a]0=not at all easy, 10=extremely easy
[b]0=not at all helpful, 10=extremely helpful
N=10. Two participants were unable to play Tetris while at work due to the limitations of their working environment (e.g., clinical duties, not having the time or space to do so).

[d]0=not at all burdensome, 10=extremely burdensome
[e]0=extremely unwilling, 10=extremely willing
[f]0=extremely unconfident, 10=extremely confident
[g]0=not at all easy, 10=extremely easy
[h]0=not at all burdensome, 10=extremely burdensome

References

1. Barlow DH, Nock M, Hersen M. Single Case Experimental Designs: Strategies for Studying Behavior for Change. 3rd edition. Pearson; 2008.

2. Tetris. The addictive puzzle game that started it all! URL: https://tetris.com/ [accessed 2024-07-02].

3. Kazdin AE. Single-case experimental designs. Evaluating interventions in research and clinical practice. Behaviour Research and Therapy [2019;117:3-17. doi:10.1016/j.brat.2018.11.015]

4. Morley S. Single Case Methods in Clinical Psychology: A Practical Guide. 1st edition. Routledge; 2017.

5. Luik AI, Iyadurai L, Gebhardt I, Holmes EA. Sleep disturbance and intrusive memories after presenting to the emergency department following a traumatic motor vehicle accident: an exploratory analysis. Eur J Psychotraumatol. 2019;10(1):1556550 [doi:10.1080/20008198.2018.1556550]

6. Tarlow KR. An Improved Rank Correlation Effect Size Statistic for Single-Case Designs: Baseline Corrected Tau. Behav Modif. 2017;41(4):427-467 [doi:10.1177/0145445516676750]

7. Vannest KJ, Parker RI, Gonen O. Single case research: Web based calculators for SCR analysis (Version 1.0) [Web based application]. College Station, TX: Texas A&M

University. Published online 2011. URL: http://www.singlecaseresearch.org/calculators/tau-u [accessed 2021-08-04]

8. Parker RI, Vannest KJ, Brown L. The Improvement Rate Difference for Single-Case Research. Exceptional Children. 2009;75(2):135-150 [doi:10.1177/001440290907500201]
