# Supplementary material for: Reducing the Number of Intrusive Memories of Work-Related Traumatic Events in Frontline Health Care Staff During the COVID-19 Pandemic: Case Series
Source: JMIR Hum Factors. 2024 Nov 18;11:e55562. doi: 10.2196/55562 (PMC11612583; doi:10.2196/55562)
Supplement: Multimedia Appendix 2 [file humanfactors_v11i1e55562_app2.pdf]

Hotspots Form

Participant ID (e.g., 001)

Today's Date (dd/mm/yyyy)

Number of work-related traumatic events the participant is having intrusive memories of:

<u>Traumatic event 1</u>

How long ago did the event take place (month, year):

Please briefly describe the worst moments of the traumatic event, e.g., an image or a sound. It is fine to summarize them in just a few words.

Brief description:

Label/name of intrusive memory:

Label/name of intrusive memory:

Label/name of intrusive memory:

Label/name of intrusive memory:

<u>Traumatic event 2</u>

How long ago did the event take place (month, year):

Please briefly describe the worst moments of the traumatic event, e.g., an image or a sound. It is fine to summarize them in just a few words.

Brief description:

Label/name of intrusive memory:

Label/name of intrusive memory:

Label/name of intrusive memory:

Label/name of intrusive memory:

Traumatic event 3

How long ago did the event take place (month, year):

Please briefly describe the worst moments of the traumatic event, e.g., an image or a sound. It is fine to summarize them in just a few words.

Brief description:

Label/name of intrusive memory:

Label/name of intrusive memory:

Label/name of intrusive memory:

Label/name of intrusive memory:

Traumatic event 4

How long ago did the event take place (month, year):

Please briefly describe the worst moments of the traumatic event, e.g., an image or a sound. It is fine to summarize them in just a few words.

Brief description:

Label/name of intrusive memory:

Label/name of intrusive memory:

Label/name of intrusive memory:

Label/name of intrusive memory:
