# Supplementary material for: Reducing the Number of Intrusive Memories of Work-Related Traumatic Events in Frontline Health Care Staff During the COVID-19 Pandemic: Case Series
Source: JMIR Hum Factors. 2024 Nov 18;11:e55562. doi: 10.2196/55562 (PMC11612583; doi:10.2196/55562)
Supplement: Multimedia Appendix 3 [file humanfactors_v11i1e55562_app3.pdf]

Intrusive memory diary

Oxford University Hospitals NHS Foundation Trust
Royal Berkshire NHS Foundation Trust
University of Oxford

Intrusive Memory Diary

Thank you for taking the time to complete the diary today.

You will be asked a few questions about **each** intrusive memory that you report since your last diary entry.

For example, if you report that you had **two** intrusive memories, the sets of questions will repeat **twice**.

Please answer the questions about one intrusive memory **at a time**.

Please contact Veronika Kubickova ([redacted]) or [redacted] if you have any questions.

Participant ID (e.g. C046)

Today's Date (dd/mm/yyyy)

Have you had any intrusive memories **since your last diary entry**?

- Yes
- No

0% 100%

Survey Powered By Qualtrics

Oxford University Hospitals NHS Foundation Trust
Royal Berkshire NHS Foundation Trust
University of Oxford

How many intrusive memories have you had **since your last diary entry**? (please enter exact numbers only, e.g. 4)

0% 100%

Survey Powered By Qualtrics

Oxford University Hospitals NHS Foundation Trust
Royal Berkshire NHS Foundation Trust
University of Oxford

Please answer these questions about **one** intrusive memory you have had since your last diary entry.

Please be as honest as you feel able to be.

Which intrusive memory did you have?

- Intrusive memory of X
- Intrusive memory of Y
- Other

0% 100%

Survey Powered By Qualtrics

Were you **at work** when you had the intrusive memory of Intrusive memory of X?

- Yes
- No

Approximate time that intrusive memory of Intrusive memory of X popped into your mind e.g. 13:45 (24h clock)

Approximately **how long** did the intrusive memory of Intrusive memory of X **bother** you for?

- <1 min
- 1-5 min
- 6-10 min
- 11-30 min
- 31-60 min
- >60 min

How **distressing** was the intrusive memory of Intrusive memory of X?

Not at all distressing — Extremely distressing

0 1 2 3 4 5 6 7 8 9 10

Intrusive memory of X

How **vivid** was the intrusive memory of Intrusive memory of X?

Not at all vivid — Extremely vivid

0 1 2 3 4 5 6 7 8 9 10

Intrusive memory of X

How much did the intrusive memory of Intrusive memory of X disrupt your **concentration**?

Not at all — A great deal

0 1 2 3 4 5 6 7 8 9 10

Intrusive memory of X

How much did the intrusive memory of Intrusive memory of X **disrupt the task** you were doing?

Not at all — A great deal

0 1 2 3 4 5 6 7 8 9 10

Intrusive memory of X

0% 100%

Oxford University Hospitals NHS Foundation Trust
Royal Berkshire NHS Foundation Trust
University of Oxford

Over the past **24 hours**, how would you rate your **sleep quality**?

- Very good
- Fairly good
- Fairly bad
- Very bad

How many times have you used the **gameplay intervention** since your last diary entry? (please enter exact numbers only, e.g. 4, or 0 if not at all)

How **accurately** do you think you completed the diary?

Not at all accurately — Extremely accurately

0 1 2 3 4 5 6 7 8 9 10

Accuracy

0% 100%

Survey Powered By Qualtrics

Oxford University Hospitals NHS Foundation Trust
Royal Berkshire NHS Foundation Trust
University of Oxford

Thank you!

0% 100%

Survey Powered By Qualtrics
