# Supplementary material for: Reducing the Number of Intrusive Memories of Work-Related Traumatic Events in Frontline Health Care Staff During the COVID-19 Pandemic: Case Series
Source: JMIR Hum Factors. 2024 Nov 18;11:e55562. doi: 10.2196/55562 (PMC11612583; doi:10.2196/55562)
Supplement: Multimedia Appendix 4 [file humanfactors_v11i1e55562_app4.pdf]

Retrospective ratings of intrusive memory characteristics and impact on functioning

OVER THE LAST WEEK:

Have you had any intrusive memories? (i.e., images of a stressful event that popped into your mind when you didn't want them to)

YES **NO**

How many intrusive memories did you have?

[None (0), some (1-4), quite a few (5-10), lots (10-20), very many (21-30), a large amount (31-50), more (more than 50)]

How distressing were your intrusive memories?

[visual scale: 0 = not at all; 10 = extremely]

How vivid were your intrusive memories?

[visual scale: 0 = not at all; 10 = extremely]

How much did they disrupt your concentration?

[visual scale: 0 = not at all; 10 = a great deal]

How much did they disrupt the tasks you were doing?

[visual scale: 0 = not at all; 10 = a great deal]

Approximately how long did your intrusive memories bother you for?

[<1min, 1-5mins, 6-10mins, 11-30mins, 31-60mins, >60mins]

How much did your intrusive memories interfere with your night's sleep?

[visual scale: 0 = not at all; 10 = a great deal]

How much have your intrusive memories affected your ability to function in your daily life?

[visual scale: 0 = not at all; 10 = a great deal]

How have your intrusive memories affected your ability to function in your daily life in the past week?

[open-ended response]
