# Supplementary material for: Reducing the Number of Intrusive Memories of Work-Related Traumatic Events in Frontline Health Care Staff During the COVID-19 Pandemic: Case Series
Source: JMIR Hum Factors. 2024 Nov 18;11:e55562. doi: 10.2196/55562 (PMC11612583; doi:10.2196/55562)
Supplement: Multimedia Appendix 5 [file humanfactors_v11i1e55562_app5.pdf]

Feedback Questionnaire: acceptability and feasibility

This questionnaire asks you about your experience of taking part in the study. We very much value both positive and negative feedback, and your answers will be used to improve this research in the future, so please answer these questions as honestly as possible.

<u>The computer game Tetris</u>

How **easy** did you find playing Tetris whilst at work?

[Likert scale from 'not at all easy = 0' to 'extremely easy = 10']

How **helpful** did you find playing Tetris whilst at work?

[Likert scale from 'not at all helpful = 0' to 'extremely helpful = 10']

How **burdensome** did you find playing Tetris whilst at work?

[Likert scale from 'not at all burdensome = 0' to 'extremely burdensome = 10']

If you were experiencing intrusive memories in the future, how **willing** would you be to play Tetris if it was offered to you as something that would help?

[Likert scale from 'Extremely unwilling = 0' to 'extremely willing = 10']

If another staff member was having intrusive memories, how **confident** would you be in suggesting playing Tetris to them?

[Likert scale from ‘Extremely unconfident = 0’ to ‘extremely confident = 10’]

Do you have any suggestions for improving Tetris as something that might help with reducing intrusive memories after a traumatic event? Please note, we will explain how this might help when we contact you again to debrief you about the study.

Do you have any other comments about using Tetris as something that might help after a traumatic event?

The study in general

How **easy** did you find taking part in the study?

[Likert scale from ‘not at all easy = 0’ to ‘extremely easy = 10’]

How **burdensome** did you find taking part in the study?

[Likert scale from ‘not at all burdensome = 0’ to ‘extremely burdensome = 10’]

If we were to roll out the study on a larger scale, how could we improve the study (over and above anything you have already mentioned)?

Do you have any other comments about the study or your experience of taking part in the study (over and above anything you have already mentioned)?
